# Neuroprotective Effects of Lindleyin on Hydrogen Peroxide-Induced Cell Injury and MPTP-Induced Parkinson's Disease in C57BL/6 Mice

**DOI:** 10.1155/2020/2938432

**Published:** 2020-02-28

**Authors:** Jin-Jie Zhang, Xiao-Rong Shi, Wen-Wen Lv, Xiao-Long Zhou, Ying-Dong Sun, Bao-Yuan Li, Xiao-Long Hu

**Affiliations:** ^1^Department of Pharmacy, Binzhou Medical University Hospital, Binzhou 256603, China; ^2^State Key Laboratory of Natural Medicines, Department of TCMs Pharmaceuticals, School of Traditional Chinese Pharmacy, China Pharmaceutical University, Nanjing 210009, China

## Abstract

Oxidative stress (OS) is a crucial factor influencing the development of Parkinson's disease (PD). Here we first reported that Lindleyin (*Lin*), one of the major components of rhubarb, possessed neuroprotective effects against H_2_O_2_-induced SH-SY5Y cell injury and MPTP-induced PD of C57BL/6 mice. The results showed that *Lin* can decrease cell death and apoptotic rate induced by H_2_O_2_ through inhibiting mitochondrial apoptotic pathway and increasing the activities of SOD, GSH-Px, and CAT as well as decreasing the level of MDA. In addition, in vivo studies showed that oral administration of *Lin* (5 or 20 mg/kg) showed significant change in motor function deficits, antioxidant enzyme activities, apoptotic pathway, and tyrosine hydroxylase expression. Our results reveal that *Lin* might be a promising anti-PD agent by reducing OS and apoptosis.

## 1. Background

Parkinson's disease (PD), which causes the disorders of movement and central nervous system (CNS) problems in older adults, is reported to be the second most common neurodegenerative disease in the world. Clinical features of PD include several motor symptoms, such as tremor, rigidity, bradykinesia, and postural instability [1]. However, the pathogenesis of PD was not fully clarified, and no animal model can reproduce all of its pathological features in human. Preclinical studies have used toxin, 1-methyl-4-phenyl-1,2,3,6-tetrahydropyridine (MPTP), to induce PD-like symptoms in animals. MPTP, a lipophilic compound that can cross the blood-brain barrier (BBB), is metabolized by monoamine oxidase B (MAO-B) into MPP^+^ to generate free radicals [2]. The symptoms, resulting from oxidative stress (OS) induced by MPTP, are similar to PD of human beings. Current drugs, including levodopa, catechol-*O*-methyltransferase inhibitors, MAO-B inhibitors, and serotonin agonists [3], can improve the symptoms but cannot prevent the progression of PD owing to a series of cascade damage incidents induced by OS [4]. Therefore, agents that primarily protect neurons against OS are important in PD treatment [5].

**Figure 1:**
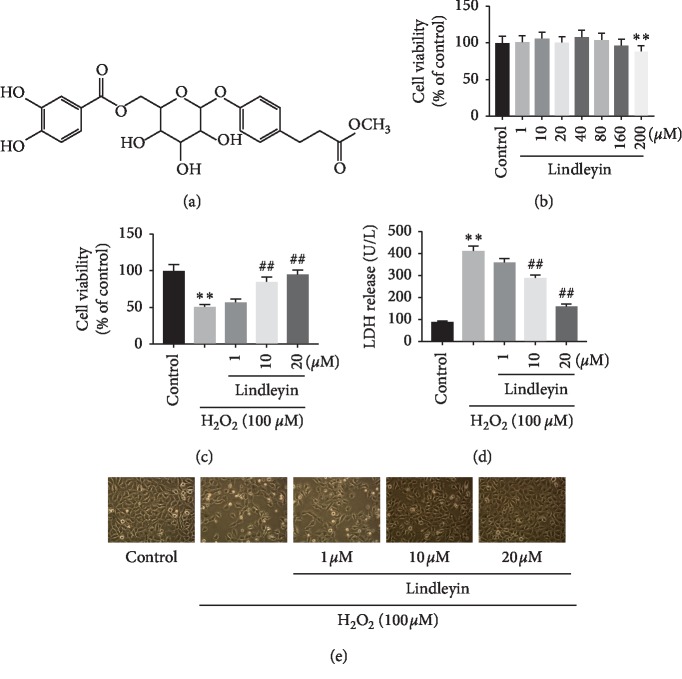
Protective effects of Lin on H_2_O_2_-induced cell injury. (a) Chemical structure of Lin. (b) Cytotoxic effects of Lin on SH-SY5Y cells. (c) Dose-dependent protective effect of pretreatment with Lin against H_2_O_2_-induced cytotoxicity in SH-SY5Y cells. (d) Protective effect of the plasma membrane damage was analyzed by LDH release. (e) The morphology of the SH-SY5Y cells followed by above treatments with an inverted microscope. The data were represented as mean ± S.E.M. of three independent experiments. ^*∗*^*P* < 0.05 and ^*∗∗*^*P* < 0.01 compared with control and ^#^*P* < 0.05 and ^##^*P* < 0.01 compared with H_2_O_2_ treatment groups.

Plant herbals are unconventional treatments widely used for several diseases. *Rhei rhizoma* (rhubarb) is a traditional Chinese herbal medicine, which commonly used as an antiphlogistic, cathartic, antipyretic, anticoagulant, and homeostatic prescription in Chinese medicine [6]. Its fresh stems and petioles could be served as vegetable, and stems are also eaten fresh [7]. Modern pharmacology demonstrated that rhubarb possesses anticancer, antioxidant, antihyperlipidemic [8–10], and antineuroinflammation properties [11]. Lindleyin (*Lin*), one of the major components of rhubarb, is shown in Figure 1(a). However, although *Lin* has been isolated for several years, the pharmacology of *Lin* is quite limited, and its antioxidant, antiapoptotic, and anti-PD properties have never been reported.

In this study, H_2_O_2_-induced SH-SY5Y cell injury and MPTP-induced PD in C57BL/6 mice were employed to investigate the neuroprotective effects of *Lin* in vitro and in vivo. This study firstly reports that *Lin* exhibits neuroprotective effects owing to its anti-OS and antiapoptotic properties both in vitro and in vivo. These findings reveal that *Lin* is a promising lead compound with potent ability of anti-OS for PD treatment.

## 2. Methods

### 2.1. Antibodies and Reagents

Lindleyin (Lin) was gained from the Chengdu Biopurify Phytochemicals Ltd. (>98.2%). Dimethyl sulfoxide (DMSO) as well as 0.25% trypsin-EDTA were bought from Amresco (Solon, OH, USA). Lactate dehydrogenase (LDH) assay kit and 3-(4,5-Dimethylthiazol-2-yl)-2,5-diphenyltetrazolium bromide (MTT) dye were bought from JianCheng Ltd. (Nanjing, China). Hoechst 33342, MPTP, and H_2_O_2_ were bought from Sigma-Chemical (St. Louis, USA). The caspase-9, caspase-3, GSH-Px, CAT, MDA, and SOD assay kits as well as 2,7′-dichlorofluorescin diacetates (DCF-DA) were acquired from Beyotime (Nanjing, China). Rabbit antibodies for TH, Bax, and Bcl-2 as well as mouse antibodies for *β*-actin, cytochrome c, and the secondary antibodies were acquired from Santa Cruz Biotechnology (Santa Cruz, USA). Rabbit antibodies for t-AKT and p-AKT were gained from Cell Signaling Technology (Inc., USA). The other used chemicals as well as reagents are all analytical grade.

### 2.2. Cell Culture

The cell lines of SH‐SY5Y human neuroblastoma had been gained from the American Type Culture Collection (ATCC, USA). Cells were cultured in DMEM high-glucose culture fluid (Sigma, USA), including 100 U/mL double antibiotics as well as 10% fetal bovine serum (Sigma, USA).

### 2.3. Cell Viability and Proliferation Assay

Protective effects of *Lin* on H_2_O_2_-induced cell injury were evaluated by MTT assay. SH-SY5Y cells were seeded within 96-well culture plates (1 × 10^4^ cells per well). After 24 hours, different concentrations (1, 10, 20 *μ*Μ) of *Lin* were added into corresponding wells to continue treatment for 2 hours, and then cells were exposed to H_2_O_2_ (100 *μ*Μ) for 24 hours. Later on, 0.5 mg/mL of MTT was put into the medium. Four hours later, the insoluble formazan crystals were dissolved by using DMSO, and the absorbance (490 nm) was detected via a microplate reader (TECAN, PRO NanoQuant).

### 2.4. Lactate Dehydrogenase (LDH) Release Assay

SH-SY5Y cells were seeded into 96-well plates and then treated with H_2_O_2_ or *Lin*. Then, the supernatants were gathered to perform the LDH assay according to the kit instructions. At last, the absorbance at 450 nm was calculated via a microplate reader (TECAN, PRO NanoQuant).

### 2.5. Hoechst 33342 Fluorescent Staining Assay

Hoechst 333342 staining assay was conducted as the previous study [12] with slight modifications. SH-SY5Y cells were treated and then washed for 3 times. Cells were stained by using 10 *μ*g/mL Hoechst 33342 dyes in the dark. The cells were imaging under a fluorescence microscope (Nikon TS-100, Japan) after being washed with cold PBS for three times.

### 2.6. Drug Administration, Animals, and PD Model's Establishment

C57BL/6 mice (male, 20–22 g) had been bought from the Model Animal Research Center of Nanjing University. Animals had been housed in a room where the temperature was controlled between 22 and 25°C and there was also a 12 : 12-hour light: dark cycle. Double distilled water had been adopted for dissolving the *Lin*. Mice had been classified randomly into five types (12 mice/cage), A group: normal control groups; B group: *Lin*-only (10 mg/kg) groups; C group: MPTP (20 mg/kg) groups; D group: MPTP (20 mg/kg) + *Lin* (10 mg/kg) groups; E group: MPTP (20 mg/kg) + *Lin* (20 mg/kg) groups. *Lin* had been preadministered intragastrically (i.g.) at 20 mg/kg or 10 mg/kg body weight every day for 12 days. Animals within Groups C and A had been administered with equivalent Double distilled water before the injection of MPTP. After the administration of *Lin* for 12 days, the intraperitoneal (i.p.) injections of MPTP (20 mg/kg) or vehicle had been administered four times to C57BL/6 mice every two hours a day.

All experiments were implemented according to the guidelines of Ministry of Health of People's Republic of China as well as the Animal Care Committee of Binzhou Medical University. All the protocols were approved by the Experimental Animal Research Committee of Binzhou Medical University.

### 2.7. Rota-Rod Test

The experiment had been implemented as what was described before and assessed by adopting a rotary rod *apparatus*. Groups A–E had been trained before for three days prior to the administration of *Lin*. The training included 3 consecutive runs which increased gradually in rpm up to a maximum 25 rpm until the mice were able to make themselves not fall from the rotary rod for 180 sec. The time on rod were tested at 4, 24, 48, and 72 hours after the last time of MPTP administration.

### 2.8. Brain Preparation

At the 12^th^ day after MPTP administration, six mice in every group were anesthetized using ether, and they were later intracardially perfused with cold 0.9% sodium chloride and 4% paraformaldehyde (PFA, PH = 7.4) in 0.1 M sodium dihydrogen phosphate. Brains were separated after fixative perfusion and then put within 4% PFA overnight at 4°C. Then, brains were transferred to a 30% sucrose solution till settle down. The SNpc and Str were serially sectioned at 40 *μ*M from cryoprotected brains in the coronal plane by adopting a freezing microtome (Leica CM3050 S, Germany), reserved within Dulbecco's PBS solution at 4°C.

### 2.9. Measurement of GSH-Px, SOD, CAT Activities, and MDA Level

This experiment was implemented as the study reported before [13] with a little modification. Cells: SH-SY5Y cells were seeded into 6-well plates (2 × 10^5^ cells/well). After treatments mentioned above, cells had been lysed on ice for half an hour by using RIPA lysis buffer. Later on, the lysed cells had been centrifuged (10, 000 g, 15 min). Then, the supernatant had been gathered for detecting the protein concentrations by adopting the BCA assay kit. Afterwards, the supernatant was subpackaged for the assays of SOD, GSH-px, and MDA level as well as CAT activities separately, in accordance with the instructions of the manufacturer. The activities of CAT and SOD as well as GSH-Px had been expressed to be % of control, and the MDA level had been expressed to be *μ*mol/mg of proteins.

Brain tissues: at the 12^th^ day after MPTP administration, six mice in every group had been anesthetized with ether and then killed to obtain brain. Afterwards, the SNpc and STR were rapidly striped and stored at −80°C. The performances of SOD, MDA level, and activities as well as GSH-Px were the same as mentioned above.

### 2.10. Western-Blot Analysis

It was performed according to the previous study [14] with slight modifications. SH-SY5Y cells were into 6-well plates. At the end of described treatments, cells were incubated with RIPA lysis buffer for half an hour and later centrifuged. The substantia nigra of each brain (*n* = 6) was separated and then conducted as mentioned above. After the protein concentration regulating to 3 mg/mL, every sample was separated on SDS polyacrylamide gel (12%). After transferring and blocking, PVDF membranes were incubated with primary antibodies (*β*-actin, Bax, caspase-9, caspase-3, and cytochrome c as well as Bcl-2, all dilution in 1 : 1000) overnight, and then the membranes were incubated by using secondary antibodies (1 : 10000). Finally, the antibody-bound proteins were detected via the enhanced chemiluminescence (ECL) method with a Chemiluminescence Apparatus (Tanon, 5500). ImageJ software was adopted for quantifying the bands' intensity.

### 2.11. Immunohistochemistry for Tyrosine Hydroxylase (TH)

DAB assay had some similarities with the previous study [15] with a little modification. In brief, brain sections were incubated with 0.6% H_2_O_2_ for 20 minutes and then washed for three times by using Tris-buffered saline (TBS). Later on, brain sections were incubated within the blocking liquid. After that, sections were incubated with primary antibody tyrosine hydroxylase (TH, 1 : 200) for 48 hours at 4°C. After being washed with TBS for four times, sections were incubated with secondary antibodies (1 : 800) for 60 minutes at room temperature. Then, sections were incubated with DAB solution in the dark for five minutes. Images were obtained with a DP72 digital camera (Nikon TS-100, Japan), and Nikon Eclipse Ts100 software was used to obtain and analyze the pictures.

### 2.12. Assays of Caspase-3 and Caspase-9

SH-SY5Y cells were seeded to 6-well plates. After treatments mentioned above, cells were collected and washed by using cold PBS and then lysed by adopting the SL-1000D ultrasonic cell disruption apparatus (Shunliu Instrument Company, China). The acquired lysate was then centrifuged, and the supernatant was used to detect the activities of caspase-9 and caspase-3. The adsorption values (405 nm) were recorded by a microplate reader (Tecan, Austria).

### 2.13. Statistical Analysis

Data was expressed as mean ± S.E.M or mean ± S.D. Analysis of variance (ANOVA) with Tukey's HSD-post hoc test procedure was adopted for determining the significance of the difference between groups, and *P* < 0.05 was regarded to be significant differences.

## 3. Results and Discussion

### 3.1. Protective Effects of Lin on H_2_O_2_-Induced SH-SY5Y Cell Injury

Hydrogen peroxide (H_2_O_2_) has been considered to be a major contributor to reactive oxygen species (ROS) generation [16]. H_2_O_2_ is easily converted into highly reactive hydroxyl radicals to attack biological pathways, leading to decreased antioxidant enzyme activities, mitochondrial dysfunction, and also apoptosis in neurons. Therefore, an H_2_O_2_-induced neuron cell injury model was established to evaluate the potential antioxidant effects of *Lin* in vitro. In the MTT assay, *Lin* at 200 *μ*M had no effect on SH-SY5Y cell viability (Figure 1(b)). The exposure of cells to H_2_O_2_ at 100 *μ*M greatly decreased cell viability to about 50 % of those in the control groups. However, the cells pretreated with *Lin* at 1, 10, or 20 *μ*M showed effectively decreased H_2_O_2_-induced cell injury compared with the H_2_O_2_-treated cells (Figure 1(c)). In addition, LDH released into cell medium was calculated, for the purpose of further investigating the protective effects of *Lin* against H_2_O_2_-induced cell injury [17]. Exposure of the cells to H_2_O_2_ at 100 *μ*M increased LDH leakage to 430.05 ± 18.32 U/L compared with the control (90.18 ± 12.11 U/L), but this value was markedly reduced to 390.8 ± 18.11, 280.42 ± 21.23, and 120.11 ± 10.21 U/L after *Lin* pretreatment at 1, 10, or 20 *μ*M, respectively (Figure 1(d)). All these results showed that *Lin* has potent protective effects against H_2_O_2_-induced cell injury.

### 3.2. Effects of Lin on H_2_O_2_-Induced Apoptosis in SH-SY5Y Cells

**Figure 2:**
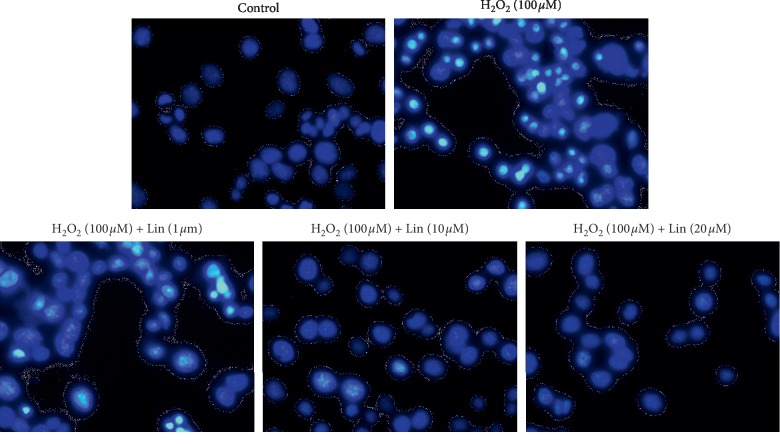
Effects of Lin on glutamate-induced nuclear condensation in SH-SY5Y cells. Cells were pretreated with Lin for 2 hours (1, 10, and 20 *μ*M) and then exposed to H_2_O_2_ for 24 hours. Representative fluorescence images were obtained after Hoechst 33342 staining in SH-SY5Y cells (100x).

Reduced number of viable cells, shrinkage, and aggregation of cell bodies occurred after treatment of H_2_O_2_ (100 *μ*M) for 24 h. By contrast, pretreatment with Lin attenuated greatly cell damage's morphological changes in a dose-dependent manner (Figure 1(e)). Furthermore, in order to investigate whether *Lin* inhibits H_2_O_2_-induced apoptosis, Hoechst 33342 staining was performed.  Figure 2 shows that nuclear condensation was presented in the cells treated with H_2_O_2_. Nevertheless, pretreatment with Lin restrained such features of apoptosis.

### 3.3. Effects of Lin on the Activities of SOD, GSH-Px, CAT, and the Degree of MDA In Vitro

**Figure 3:**
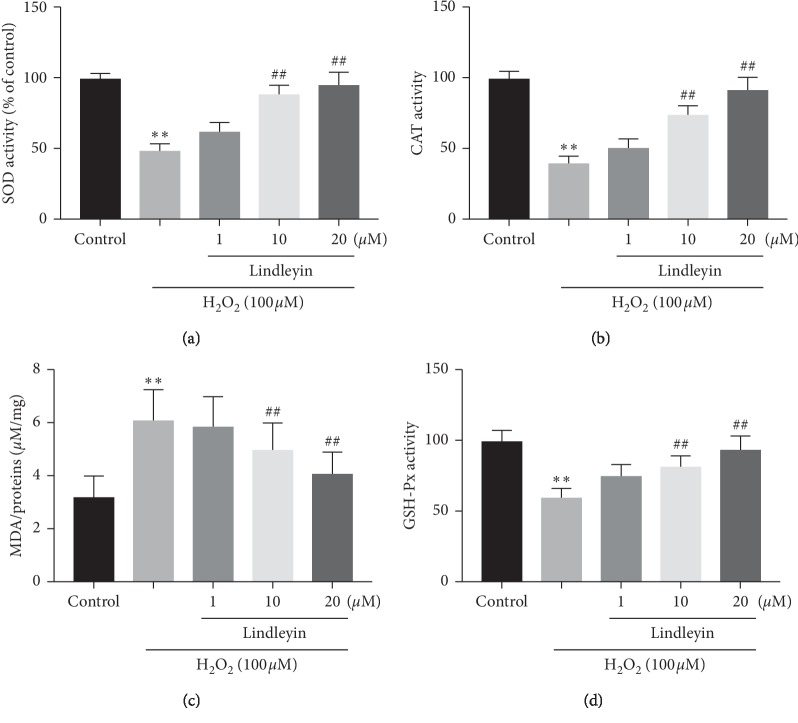
Effects of Lin on antioxidant enzymes. (a) SOD activities. (b) CAT activities. (c) GSH-Px activities. (d) MDA levels. Cells were incubated with different concentrations of Lin for 2 hours before being exposed to 100 *μ*M H_2_O_2_ for 24 hours. The data were represented as mean ± S.E.M. of three independent experiments. ^*∗*^*P* < 0.05 and ^*∗∗*^*P* < 0.01 compared with control and ^#^*P* < 0.05 and ^##^*P* < 0.01 compared with H_2_O_2_ treatment groups.

OS and free radical production play critical roles in modulating redox reactions and contributing ROS, which is the main assailant in neurodegeneration. Evidence demonstrated that OS also plays a critical role in the regulation of the biochemical changes regarding neurodegenerative diseases [18]. OS is inhibited by antioxidant enzymes, whose activities are decreased in neurodegenerative disorders [19]. The present results showed that H_2_O_2_ led to an imbalance in the antioxidant defense system, which further reduced the activities of GSH-Px and CAT as well as SOD and improved the MDA degree. However, treatment with *Lin* dramatically improved the activities of these antioxidant enzymes and reduced the level of MDA (Figures 3(a)–3(d)). These data suggested that *Lin* can decrease H_2_O_2_-induced OS via decreasing the levels of intracellular lipid peroxidation as well as the activities of antioxidant enzyme.

### 3.4. Effects of Lin on the Mitochondrial Apoptotic Pathway In Vitro

**Figure 4:**
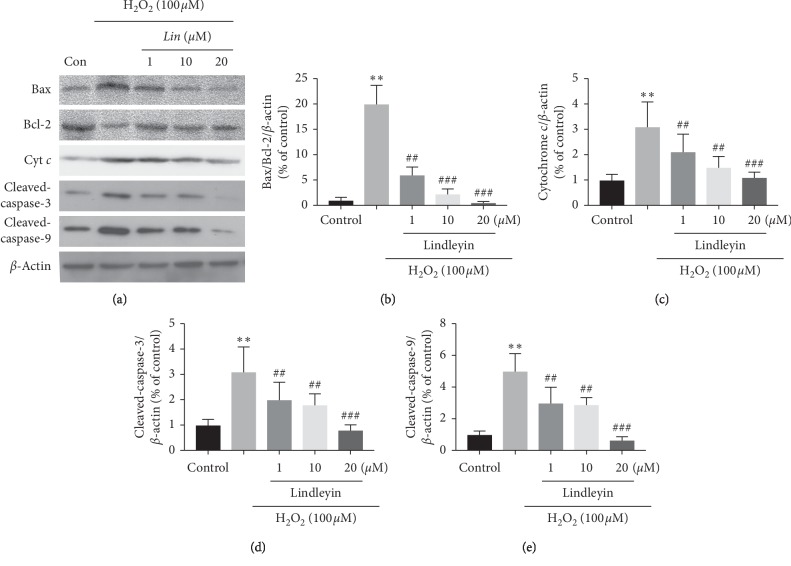
Western-blot analysis of apoptosis-related proteins. (a) The original bands of each protein. (b) The ratio of Bax/Bcl-2. (c) The quantitative analysis of cytochrome *c*. (d) The quantitative analysis of cleaved-caspase-3. (e) The quantitative analysis of cleaved-caspase-9. The data were represented as mean ± S.E.M. of three independent experiments. ^*∗*^*P* < 0.05 and ^*∗∗*^*P* < 0.01 compared with control and ^#^*P* < 0.05 and ^##^*P* < 0.01 compared with H_2_O_2_ treatment groups.

Mitochondria exhibits a pivotal role in apoptosis induced by OS [20]. Mitochondria-related apoptotic proteins have been related with H_2_O_2_-induced cytotoxicity in neuronal loss [21]. The antiapoptotic factor Bcl-2 restrains the release of cytochrome *c* [21, 22] and the proapoptotic factor Bax promotes the release of cytochrome *c* [23]. Cytochrome *c* then triggers the activation of caspase-3 and caspase-9, leading to cell apoptosis [24]. The present results showed that *Lin* can improve the ratio of Bcl-2/Bax (Figure 4(a)) and reduce the release of cytochrome *c* (Figure 4(b)), and the expressions of caspase-9 (Figure 4(c)) and caspase-3 (Figure 4(d)). These results suggest that *Lin* provides neuroprotective effects through inhibiting the mitochondrial apoptotic pathway.

### 3.5. Effects of Lin on MPTP-Induced Mice Model of PD

**Figure 5:**
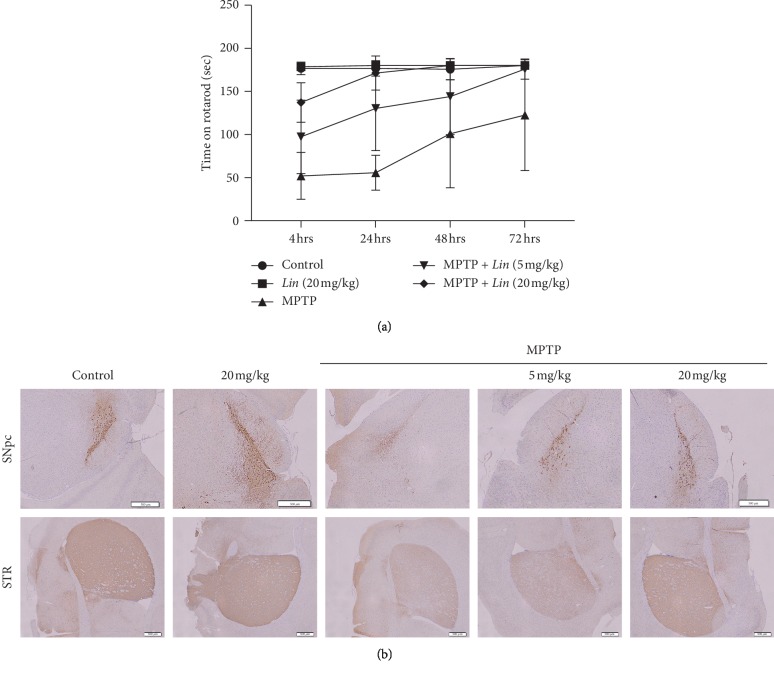
Lin alleviated functional disturbance in the MPTP-induced PD model. (a) MPTP-induced motor disability was evaluated with the rotarod test. All mice were pretrained for 3 days on the rotarod. The MPTP-treated group had significantly poorer rotarod performance than treatment normal controls, but morin pretreatment (5 or 20 mg/kg) before MPTP improved rotarod performance vs. the MPTP-treated group at 4, 24, 48, and 72 hours after final MPTP treatment. (b) Lin attenuated MPTP-induced dopaminergic neuronal loss in Str and SNpc. TH immunostaining analyses were performed on SNpc and Str.

Although the pathogenesis of Parkinsonism was still unclear, dopaminergic neurons death in the substantia nigra pars compacta to oxidative stress had been considered to be one of the main causes in PD [25]. The lipid peroxidation product could be increased by MPTP, and, therefore, the effects of *Lin* against oxidative stress in MPTP-induced mice model were evaluated. Results suggested that MPTP notably impaired performance compared with the normal controls. However, *Lin* significantly alleviated the functional impairment (Figure 5(a)). The early loss of tyrosine hydroxylase (TH) activities followed by a decline in the level of TH protein contributes to dopamine deficiency. The reduction in TH-positive neurons in response to MPTP treatment has been considered to be the most significant at medial levels of the SNpc [12]. MPTP group revealed significantly less TH-positive cells than the normal controls in both Str and SNpc. In contrast, pretreatment with *Lin* at 5 or 20 mg/kg significantly prevented this loss in Str (Figure 5(b)) and SNpc, indicating that *Lin* decreases the dopaminergic neuronal loss induced by MPTP.

### 3.6. Impacts of Lin on the Activities of CAT, GSH, and SOD as well as the Degree of MDA In Vivo

**Figure 6:**
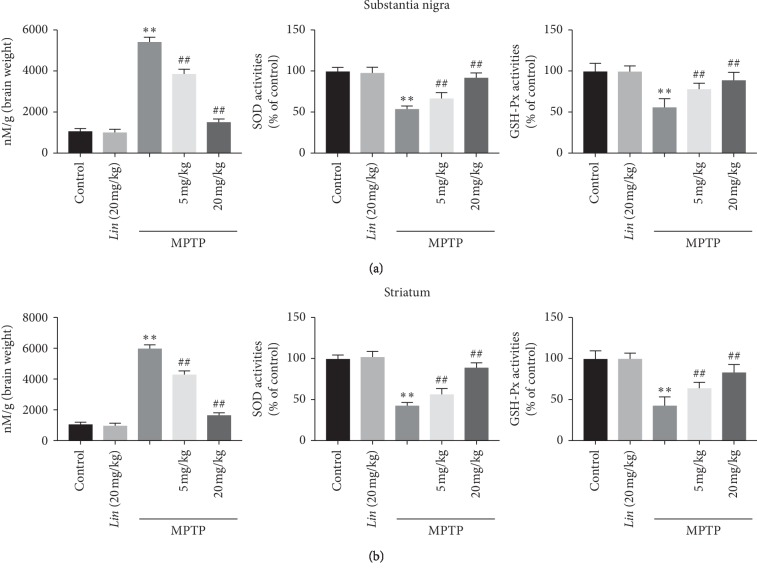
The effects of *Lin* on antioxidant enzymes in vivo. (a) The activities of SOD, GSH, and the level of MDA in SNpc. (b) The activities of SOD, GSH, and the level of MDA in STR. The data were represented as mean ± S.E.M. (*n* = 10 mice/group). ^*∗*^*P* < 0.05 and ^*∗∗*^*P* < 0.01 compared with control and ^#^*P* < 0.05 and ^##^*P* < 0.01 compared with MPTP treatment groups.

The mentioned results have demonstrated that *Lin* can reduce oxidative stress through improving the activities of CAT, GSH, and SOD, and reducing the MDA degree in vitro. Therefore, whether *Lin* can also show the same biological activities in vivo should be further confirmed. The outcomes indicated treatment with MPTP significantly decreased the activities of GSH, SOD, and CAT and increased the MDA degree in both SNpc and STR (Figures 6(a) and 6(b)). However, oral administration of *Lin* (5, and 20 mg/kg) can reverse this tendency, which were the same as in vitro study, indicating that *Lin* also has antioxidative effects in vivo.

### 3.7. Impacts of Lin on the Expressions of Bcl-2 as well as Bax, and the Activities of Caspase-9 and Caspase-3 In Vivo

**Figure 7:**
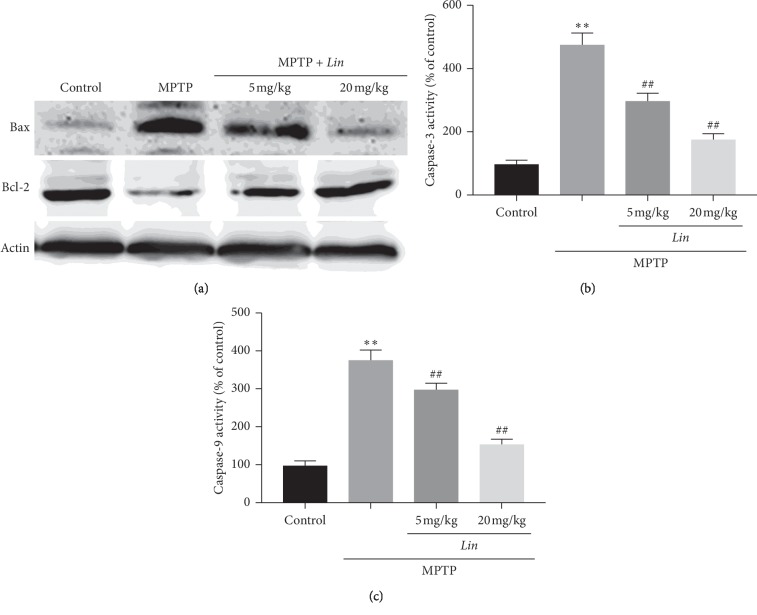
Western-blot analysis of apoptosis-related proteins and activities of caspases family. (a) The original bands of Bax and Bcl-2 proteins. (b) The activity of caspase-3. (c) The activity of caspase-9. The data were represented as mean ± SD (*n* = 6). ^*∗∗*^*P* < 0.01 compared with control, and ^##^*P* < 0.01 and compared with MPTP groups.

The mentioned results have demonstrated that *Lin* can reduce the apoptosis induced by H_2_O_2_ in vitro. Therefore, in order to confirm that *Lin* can also reduce the apoptosis induced by MPTP in vivo, western-blot analysis was adopted for detecting the expressions of Bcl-2 as well as Bax, and the activities of caspase-9 as well as caspase-3 in vivo. Outcomes presented the notion that MPTP greatly improved the expression of Bax and reduced the expression of Bcl-2 and the activities of caspase-3 and caspase-9 in SNpc. However, oral treatment with Lin can inhibit the expressions or activities of these apoptosis-related proteins in a dose-dependent manner (Figures 7(a)–7(c)). These results demonstrated that Lin can protect TH neuronal loss through inhibiting apoptosis.

## 4. Conclusions

In summary, we firstly evaluated the effects of *Lin* on H_2_O_2_-induced oxidative stress in SH-SY5Y cells as well as MPTP-induced PD-like symptoms in C57BL/6 mice. The results suggested that *Lin* effectively inhibited the cells injury induced by H_2_O_2_ through antioxidant and antiapoptosis pathways both in vitro and in vivo. Notably, *Lin* obviously prevented MPTP-induced PD-like pathologies in mice at an inferior dose, including behavioral testing and TH expression. The represented report suggested the possibilities that *Lin* should be considered as one of the antioxidant agents for the development of a therapeutic agent for PD.

## Data Availability

The data used to support the findings of this study are available from the corresponding author upon request.

## Abbreviations

PD: Parkinson's disease
OS: Oxidative stress
DMEM: Dulbecco's modified Eagle's medium
DAB: Diaminobenzidine
DMSO: Dimethyl sulphoxide
MPTP: 1-Methyl-4-phenyl-1,2,3,6-tetrahydropyridine
MTT: 3-(4, 5-Dimethylthiazol-2-yl)-2, 5-diphenyltetrazolium bromide
SOD: Superoxide dismutase
MDA: Malondialdehyde
MAO-B: Monoamine oxidase B
LDH: Lactate dehydrogenase.
